# Prevalence and risk factors of suicidal ideation amongst unaccompanied young refugees: a machine learning approach

**DOI:** 10.1007/s00787-025-02828-0

**Published:** 2025-09-12

**Authors:** Jacob Keller, Jenny Eglinsky, Maike Garbade, Elisa Pfeiffer, Paul L. Plener, Rita Rosner, Thorsten Sukale, Cedric Sachser

**Affiliations:** 1 Clinical Child and Adolescent Psychology, Institute of Psychology, University of Bamberg, Kapuzinerstraße 32, 96047 Bamberg, Germany; 2 Department of Child- and Adolescent Psychiatry, Psychosomatics and Psychotherapy, Ulm University, Ulm, Germany; 3 German Center for Mental Health (DZPG), Partner Site Ulm, Ulm, Germany; 4 Department of Clinical Psychology and Child and Adolescent Psychotherapy, Catholic University Eichstätt-Ingolstadt, Eichstätt, Germany; 5 Department of Child and Adolescent Psychiatry, Medical University of Vienna, Vienna, Austria; 6 Department of Psychology, Catholic University Eichstätt-Ingolstadt, Eichstätt, Germany; 7 Praxis Sukale & Markthaler, Krumbach, Germany

**Keywords:** Suicidal ideation, Unaccompanied young refugees, Prevalence, Risk factors, Machine learning

## Abstract

**Background:**

Suicidality is a major public health concern worldwide. Evidence on the prevalence and risk factors of suicidality amongst unaccompanied young refugees (UYRs), a population already at risk for mental health disorders, is scarce.

**Methods:**

Given the complexity of individual risk factor constellations influencing suicidality, machine learning (ML) methods offer a statistical approach that can detect complex relations within the data. Four ML classifiers, (logistic regression (LR), random forest (RF), support vector machines (SVM), and extreme gradient boosting (XGB)) were trained on a dataset of *n* = 623 UYRs (M_age_=16.77, SD = 1.34, range: 12–21), retrieved from the large-scale randomized controlled trial *Better Care* to predict suicidal ideation. Features used in the classifiers were age, gender, asylum status, having contact with the family, and whether parents are alive as well as clinically elevated post-traumatic stress symptoms (PTSS), depressive symptoms and past suicide attempts. The classifiers were then tested on the independent dataset of *n* = 94 UYRs (M_age_=16.31, SD = 2.03, range: 5–21) retrieved from the screening tool *porta* project to examine their predictive performance.

**Results:**

The prevalence of past-week suicidal ideation in the combined sample of *N* = 717 was 18.13%. All classifiers yielded good predictive performance (accuracy 0.734–0.840, sensitivity 0.857, AUC 0.853–0.880). The most relevant features were past suicide attempts, PTSS and depressive symptoms as risk factors, and having a living mother as protective factor.

**Conclusions:**

Suicidal ideation is prevalent amongst UYRs, and using ML approaches, the classifiers were able to classify roughly 85% of the cases with suicidal ideation in the past week correctly as suicidal. Building on the findings of this study, screening for suicidality could be further improved by implementing ML classifiers in the assessment to highlight potential at risk cases early, and suitable interventions be developed.

## Introduction

Suicidality among adolescents is a central public health concern and a leading cause of death in this age group [1]. Whilst being highly prevalent, suicidal ideation, meaning thoughts about ending one’s life and behaviors, often go undetected by parents, teachers, and health care professionals [2]. Building concise frameworks for suicidal ideation to aid detection and early interventions remains challenging due to the highly individual nature of problem constellations leading to suicidal ideation [3].

Unaccompanied young refugees (UYRs) are often exposed to multiple potentially traumatic experiences [4]. Next to these experiences, postmigration stressors in the host country (e.g. discrimination, the asylum process or lack of language skills) have been shown to increase symptom severity of depressive symptoms and post-traumatic stress symptoms [5–7]. A high prevalence for post-traumatic stress disorder (PTSD) amongst UYRs was found in a recent systematic review of studies concerning UYRs living in Europe [4]. Prevalence rates found in cross-sectional studies for (PTSD) ranged from 4.6% up to 43%, with the high range regarding PTSD being explained by the highly varying study samples [4]. Three studies included in the review investigated suicidal behavior [4.] Two of the studies found higher rates of suicide attempts and suicide deaths among UYRs compared to the general population [4]. The third study reported no suicide deaths amongst a population of UYRs [4].

In the studies comparing accompanied with unaccompanied young refugees, being unaccompanied was found to be a risk factor for more severe mental health difficulties, with UYRs being at risk for a higher trauma load than accompanied young refugees [4]. Aligning with this, studies investigating social support reported higher levels of symptom severity for UYRs with lack of social support [4.] It was found that UYRs receive the most social support from their families and being in contact with the family affected the reception of social support provided in the host country [4]. The ability of social support to potentially mitigate the impact of stress on mental health, underlines its relevance as a potential protective factor [4]. Additionally, being older or having an insecure asylum status were identified as risk factors in several studies [4]. It was found that older UYRs receive less support in the host country by professionals than younger UYRs [4]. Furthermore, UYRs awaiting a decision on their asylum application presented higher rates of PTSD, depression and anxiety [4].

Another study investigating a population of *N =* 75 UYRs living in Norway reported a prevalence of 11% for suicidal ideation [8]. This study was also part of a systematic review by Jin et al. [9] investigating depression and suicidality amongst child and adolescent refugees mainly living in the US, Australia and Norway. In the review, an increased risk for depression and suicidality amongst refugee adolescents compared to the host countries’ general population was found [6]. Rates for suicidal behavior ranged from 1.79 to 57.9% in varying samples of UYRs and accompanied young refugees. Aligning with previous findings [4], higher rates of suicidal behavior were found among refugee adolescents compared to the general population [9]. The study further highlights that being accompanied compared to being a UYR is deemed to be a protective factor for depressive symptoms [9]. A caveat mentioned by the authors was the difficulty in differentiating between the influences of depression and PTSD on mood symptoms and suicidality due to the variety of assessment tools used across the studies [9]. A recent study concerning the effectiveness of a short-term group-based trauma-focused intervention for UYRs found a prevalence of 44% for self-harm and suicidal ideation amongst a population of UYRs with post-traumatic stress symptoms (PTSS) living in Germany [10]. This further underlines the potential link between PTSS and suicidal ideation.

With only a few studies reporting on suicidal ideation in the population of UYRs at all, reporting high variances, and building on comparably small samples, further research is needed to investigate the prevalence of suicidality and potential risk and protective factors.

Creating concise models for suicidal ideation is challenging due to the complex interplay of individual social, biological and psychological factors influencing suicidal ideation [3, 11]. Bringing together the multitude of intrapersonal factors like personality traits, psychopathology, social environment and genetics that also influence each other further complicates model creation [11]. Machine learning (ML) approaches, although relatively novel in psychological research, provide a suitable methodology to take on this challenge and have already proven themselves to be viable in predicting suicidal ideation [12, 13]. ML algorithms can be used to learn associations between cases in a dataset and then use this learned information to classify new cases into learned classes [14]. A classifier trained to distinguish cases with and without suicidal ideation, would learn the characteristics of cases with ideation, presented in the data. Based on the learned knowledge how cases with suicidal ideation are presented, the classifier could then be used on a new dataset to identify cases with suicidal ideation. To classify these cases, the classifier compares the presented cases with the learned information how suicidal ideation cases are characterized [14]. While studies building their models on electronic health records already provided promising prediction models [15, 16], studies relying on a more general population of adolescents are scarce [17].

Building on this predictive modeling approach and using clinically evaluated symptom measures, as well as social factors, this study aims to investigate the prevalence of suicidal ideation and potential risk and protective factors in a population of UYRs. To also investigate the link between suicidal ideation and social factors and to gain further knowledge about their relation, factors like asylum status, age, gender and information about the family will be included in the ML models. Previous research highlighted the protective abilities of social support and identified a link between asylum status, age and higher psychological stress [4]. Using these factors in ML algorithms could provide meaningful insights into their relations. Potentially enabling an early detection of at-risk cases and informing interventions to prevent suicidal ideation. By using four different supervised machine learning classifiers, namely lasso logistic regression (LR), random forests (RF), support vector machines (SVM), and extreme gradient boosting (XGB), suicidal ideation will be predicted, and risk factors will be assessed regarding their relevance. The classifiers will be trained on data retrieved from the *Better Care* study [18], and out-of-sample performance will be validated on a dataset retrieved from the *porta* project [19]. Models will be evaluated using accuracy, sensitivity, specificity, and the area under the curve (AUC) extracted from the Receiving Operator Characteristic (ROC). While machine learning approaches have recently been used to predict suicide attempts in this age group [8], this is the first study applying ML techniques on a sample of UYRs while also using clinically assessed suicidal ideation as the outcome variable.

## Methods

### Sample

The training sample was drawn from the *Better Care* Project study sample at baseline assessment [18]. The *Better Care* project is a multi-center, large-scale, cluster-randomized controlled trial investigating the efficacy of a stepped-care treatment approach for UYRs in Germany [18]. Complete data from *n* = 623 UYRs living in child welfare facilities in Germany gathered between 2019 and 2023 were used for the model training. To validate the models, complete data of *n* = 94 UYRs gathered between 2018 and 2024 was retrieved from *porta* [19]. *porta* is an online screening platform hosted by the university clinic Ulm for adolescent refugees where healthcare professionals and social workers can access several mental health screening tools [19]. These tools are provided as self- and proxy reports [19]. For this study, only self-reported data was used. The studies involving human participants were reviewed and approved by ethics committees at Ulm University (*porta*: No.159/16) and (*Better Care*: No. 243/19) and at the Catholic University of Eichstätt-Ingolstadt (*Better Care*: No. 004–19). Written informed consent to participate in this study was provided by the participants and their legal guardians if necessary.

In the training sample, participants were, on average, 16.77 (SD = 1.34, range: 12–21) years old, with 9.3% identifying as female. Participants mainly originated from Afghanistan (42.69%), Syria (17.01%), and Somalia (6.9%).

In the test sample, participants were an average of 16.31 years old (SD = 2.03, range: 5–21), with 12.12% identifying as female. Participants mainly originated from Afghanistan (42.42%), Syria (15.15%), and Guinea (7.07%). Sociodemographic information is depicted in Table 1.

**Table 1:** Sample characteristics of the better care and Porta samples

	Better Care Sample *n* = 623	porta Sample *n* = 94	Combined Sample *N* = 717
	n (%)	n (%)	n (%)
Secure asylum status	119 (19.10%)	17 (18.08%)	136 (18.96%)
Active schooling	550 (88.28%)	48 (51.06%)	598 (83.40%)
Mother alive	464 (74.47%)	62 (65.95%)	526 (73.36%)
Father alive	344 (55.21%)	53 (56.38%)	397 (55.36%)
In contact with parents	464 (74.47%)	56 (59.57%)	520 (72.52%)
PTSS^1^	270 (43.33%)	68 (72.34%)	338 (47.14%)
Depression^2^	260 (41.73%)	51 (54.25%)	311 (43.37%)

^1^Clinically elevated post-traumatic stress symptoms, according to the respective cutoff value,

^2^Clinically elevated depressive symptoms, according to the respective cutoff value

### Measures

Data was collected using self-reports in the adolescent’s preferred language if a translation was available. If available, interpreters supported the assessment with translating questions.

#### Suicidal ideation

In the training sample, UYRs who positively screened for suicidal ideation on the ninth item of Beck’s Depression Inventory (ranging from “I don’t have thoughts of killing myself” to “I would kill myself if I had the chance”) [20], during the questionnaire assessment, were further interviewed for suicidal ideation. In the interview the Columbia Suicide Severity Rating Scale (C-SSRS), developed by Posner et al. [21], was used. To assess past-week severity of suicidal ideation, the first five items of the C-SSRS were used [21]. These items cover ideation severity on five levels, ranging from “Wish to be Dead” to “Active Suicidal Ideation with Specific Plan and Intent” [21]. Prior suicide attempts were assessed with one item and coded as 1, indicating a prior suicide attempt, and 0, indicating no prior suicide attempt [21]. Both assessment tools have previously shown high internal consistency in samples of adolescents [22, 23] Notably, only adolescents interviewed for suicidal ideation were screened for past attempts. Past week suicidal ideation as assessed with the C-SSRS was aggregated into a binary variable to enable its use in the ML classifiers. Any indication of ideation in the past week was coded as 1, no indication was coded as 0.

In the test sample, suicidal ideation and past attempts were assessed using the “Self-injurious thoughts and behaviors interview” [24] in the German version in the form of a self-report questionnaire [25]. Suicidal ideation was coded into a binary variable using the seventh item of the SIT-BI, which assesses how many times suicidal ideation was present in the past week [24].

#### Post-traumatic stress symptoms and trauma load

PTSS were assessed using the German version of the “Child and Adolescent Trauma Screen” (CATS) [26] in the test sample, and the “Child and Adolescent Trauma Screen Version 2” (CATS-2) [27] in the training sample. The CATS and the CATS-2 consist of a checklist containing 15 potentially traumatic events (PTEs). Subsequently, 20 items assessing PTSS are scored on a 4-point Likert scale ranging from “never” to “almost always” followed by five items assessing psychosocial functioning [26, 27]. The PTSS items are summed up to build the PTSS sum score, with a cutoff value of 21 or higher for the CATS and a value of 25 or higher for the CATS-2, indicating probable PTSD [27]. Internal consistency for PTSS in the training and test sample was good (training sample *ω* = 0.92, test sample *ω* = 0.93). PTSS were then coded into a binary variable, with 1 indicating a score above or equal to the cutoff value and 0 indicating a score beneath the cutoff. Trauma load was aggregated from the PTE checklist and used as a sum score.

#### Depressive symptoms

Depressive symptoms were assessed using the Patient Health Questionnaire [28]. The PHQ-9 measures depression via nine items, using a 4-point Likert scale ranging from “not at all” to “nearly every day” [28]. A cutoff score of 10, indicating the likely presence of major depression, was used for this study [28]. The internal consistency for the PHQ-9 in the training and test samples was good (training sample *ω* = 0.85, test sample *ω* = 0.90). Depressive symptom severity was also coded into a binary variable based on the cutoff.

#### Machine learning

This study compared four ML classifiers, logistic regression with lasso regularization, random forest, support vector machines, and extreme gradient boosting, in terms of their ability to predict suicidal ideation. All four algorithms have already been used to predict suicidal ideation and showed good predictive performance [12, 13, 29]. Analyses were run in R (Version 4.2.3) [30], using the packages caret [31], glmnet [32], pROC [33] and psych [34].

Logistic regression with the least absolute shrinkage and selection operator (LASSO) regularization was used to predict a binary category by using predictor variables [35]. The regularization was carried out using the hyperparameter *λ* (lambda), which defines a penalty leading to fewer contributing coefficients to be set to zero [35]. For this study, the decision threshold was set to *p* = 0.5. Compared to other ML classifiers, Lasso regression provides more interpretable information regarding the used features [29].

Using bootstrap aggregation, random forest (RF) classification builds on the decision tree logic [35]. In a decision tree, data is iteratively split into subsets called nodes [14]. The goal is to create nodes using the predictor variables that mostly contain one class and are, therefore, relatively pure [14]. In RF classification several decision trees with varying constraints are created and then aggregated, which improves classification [13–35]. RF classifiers can be tuned for variables tried at each split and the number of different trees that will be created [35].

In support vector machine (SVM) classification, the algorithm tries to fit a hyperplane based on support vectors into the higher-dimensional feature space that best separates the two classes [37]. Support vectors are the points most suitable for separating the two classes [37]. SVM can be tuned using the hyperparameter c or cost, imposing a misclassification penalty [35].

Extreme gradient boosting (XGB) also builds on the decision tree logic [35]. In XGB, trees are built sequentially, meaning that each tree is built using information from the previously built tree [35]. This enables the classifier to learn better in areas where the performance was weaker, gradually improving performance [38]. XGB can be tuned via the number of trees grown, the learning rate, and the number of splits contained in each tree [38].

As predictors, also called features in the ML context, clinically elevated PTSS and depressive symptoms coded into binary variables indicating the likely presence of PTSD and depression, age, gender, and the sociodemographic variables presented in Table 1 will be used. Given that no missing data occurred in the data, no methods for adjusting for missing data were employed.

To prevent overfitting, k-fold cross-validation with *k* = 10 was implemented into the model training process [38]. With k-fold cross-validation, data is randomly split into smaller datasets, also called folds [38]. Each fold is then used as a test set, with a model being trained on the remaining folds [38]. Model fit criteria are extracted for each fold, and overall model performance is calculated by averaging the extracted fit criteria [38]. During training, classifiers were tuned for their respective hyperparameters via grid search, which allows to test a range of values for each hyperparameter. Hyperparameters that produced the best accuracy were used for the final models. Tuning results and the respective hyperparameters used in the final models are presented in Table 2.

**Table 2:** Tuned hyperparameters

Classifier	Hyperparameters
LR^1^	Lasso regularization, *λ* = 0.01
RF^2^	Variables tried per split = 3, Number of trees = 1000
SVM^3^	linear kernel, c = 0.001
XGB^4^	Number of trees = 500, tree length = 3, d = 0.01, minimum loss reduction = 0, subsample ratio of columns = 0.8, minimum sum of instance weight = 3, subsample percentage = 1

^1^Logistic regression, ^2^Random forest, ^3^Support vector machines, ^4^Extreme gradient boosting

Models were evaluated for their predictive ability using accuracy, sensitivity, specificity, and the area under the curve (AUC) metric retrieved from the ROC curve. The AUC describes the classifier’s ability to distinguish classes, ranging from 0.5 to 1, with 0.5 indicating that the classifier’s prediction is not better than chance [38].

## Results

The prevalence rate for past week suicidal ideation in the combined sample was *n* = 130 (18.13%), *n* = 123 (19.74%) in the training sample, and *n* = 7 (7.44%) in the test sample. Self-reported past suicide attempts were reported by overall *n* = 50 (6.97%), *n* = 30 (4.81%) in the training sample, and *n* = 20 (21.27%) in the test sample.

Using *X*^*2*^ tests of difference revealed significant differences regarding suicidal ideation *X*^*2*^(1, *N* = 717) = 30, *p <* 0.01 and past attempts *X*^*2*^(1, *N* = 717) = 32.02, *p <* 0.001 between the training and test sample. After tuning, the final models were used to predict the test set and showed good predictive ability, with all models classifying six (85.71%) of the seven cases with suicidal ideation correctly. The best-performing models were the tree-based classification models XGB (Accuracy = 0.84, 95% CI: 0.75, 0.91, AUC = 0.88, 95% CI: 0.64, 0.93) and RF (Accuracy = 0.84, 95% CI: 0.75, 0.91, AUC = 0.87, 95% CI: 0.64, 0.94) with the highest accuracy and AUC.

These two models also had the highest specificity, meaning that they were not only able to recognize suicidal ideation cases correctly but also performed well in classifying cases without suicidal ideation correctly. The accuracy, sensitivity, specificity, and AUC values are presented in Table 3.

**Table 3:** Model results

Classifier	Accuracy [95% CI]	Sensitivity [95% CI]	Specificity [95% CI]	AUC^1^ [95% CI]
LR^2^	0.734 [0.63, 0.82]	0.857 [0.48, 0.97]	0.724 [0.62, 0.80]	0.867 [0.7, 0.99]
RF^3^	0.840 [0.75, 0.91]	0.857 [0.48, 0.97]	0.839 [0.74, 0.90]	0.875 [0.64, 0.94]
SVM^4^	0.744 [0.64, 0.83]	0.857 [0.48, 0.97]	0.735 [0.63, 0.81]	0.853 [0.7, 0.99]
XGB^5^	0.840 [0.75, 0.91]	0.857 [0.48, 0.97]	0.839 [0.74, 0.90]	0.880 [0.64, 0.93]

^1^Area under the curve, ^2^Logistic regression, ^3^Random forest, ^4^Support vector machines, ^5^Extreme gradient boosting

Table 4 presents each model’s five most relevant features, ranging from most to least important. Across all models, past suicide attempts were the most relevant feature for predicting suicidal ideation. PTSS and depressive symptoms were also highly relevant features over all models, followed by whether the mother was alive or not, present in three models and trauma load, present in one model. Age was also relevant in two of the four models. For the logistic model, odds ratios are also reported to enable interpretation. Attempted suicide in the past (OR = 34.91) increased the risk for suicidal ideation substantially. While PTSS (OR = 3.86) and depressive symptomology (OR = 2.86) also increased the risk for suicidal ideation, having a living mother (OR = 0.57) and contact with the family decreased (OR = 0.8) the risk for suicidal ideation.

**Table 4:** Most relevant features

Model	LR^1^ (OR^2^)	RF^4^	SVM^5^	XGB^6^
Feature 1	Past attempt (34.918)	Past attempt	Past attempt	Past attempt
Feature 2	PTSS^3^ (3.860)	Trauma Load	Living mother	Depression
Feature 3	Depression^3^ (2.866)	PTSS	PTSS	PTSS
Feature 4	Living mother (0.573)	Depression	Depression	Living mother
Feature 5	Family Contact (0.801)	Age	Age	Trauma Load

^1^Logistic regression, ^2^Odds ratio, ^3^Clinically elevated, ^4^Random forest, ^5^Support vector machines, ^6^Extreme gradient boosting

## Discussion

The aim of this study was to investigate the prevalence of suicidal ideation amongst a population of UYRs while also focusing on risk and protective factors. This study revealed that suicidal ideation is prevalent among UYRs, and utilizing ML methods to predict suicidal ideation yielded promising results, giving reason to further investigate the potential of these novel approaches for clinical use. The findings of this study regarding suicidal ideation amongst UYRs are in line with the findings of Jensen and colleagues [8], although a slightly higher prevalence rate of 18% was found in this study. Past suicide attempts have often been reported as major risk factors for further suicidal behavior [40] and our results further underline this finding also in a sample of UYRs. Comparing the rates of past attempts of UYRs in this study with the general population of German adolescents, the rate in this study falls within the range of reported rates for past attempts within German adolescents [41, 42]. In this study, 7% of UYRs reported a past attempt, while prevalence rates for past attempts among German adolescents were found to be around 6.5-9% [41, 42].

This study also found significant differences in reported prevalence of suicidal ideation and past suicide attempts between the training and the test set (*p* < 0.01, *p* < 0.001). This could be explained by the varying assessment modalities between the two samples. In the training sample, suicidal ideation was screened during the self-report questionnaire assessment after which past week suicidal ideation and past attempts were further investigated in an interview setting using the C-SSRS. In the test sample, suicidal ideation and past attempts were assessed via the SIT-BI self-report questionnaire contained in the *porta* assessment tool. The significant differences could be explained by the different assessment modalities and contexts and relating factors like trust in healthcare professionals. A systematic review on UYRs’ perception of mental health services suggests, that trust next to cultural factors could influence the willingness to disclose mental health struggles, especially if they fear that the information could be used against them [43]. With the study setting of the *Better Care* project and in which external study personnel interviewed the participants, potential distrust towards the study personnel might have influenced the willingness to disclose about suicidal ideation and past attempts. In comparison, assessment via *porta* was mainly initiated via caregivers in a familiar context and suicidal ideation and past attempts were assessed via self-report questionnaire only. With no data available on the specific assessment setting in which UYRs were screened for mental health symptoms via *porta*, the potential bias of the assessment setting could not be controlled for.

Regarding risk factors for suicidal ideation, this study supports previous findings, especially the presence of PTSS and depressive symptoms increasing the risk for suicidal ideation [4, 10]. The primary contribution of ML in this context lies not in identifying new risk factors per se, but in its ability to model complex underlying relations and interactions among multiple variables, which might be overlooked by traditional statistical approaches. Building on this, ML approaches allow for an individualized prediction with potentially higher accuracy, based on the individual risk and protective factor structure, which could benefit the detection of at-risk cases in the context of clinical screenings. Daniel-Calveras and colleagues [4] also suggest that social support plays an important role in mitigating negative consequences of stress on mental health. Although social support was not directly assessed in this study, broader social environment factors, like having a living mother and contact with the family lowered the risk for suicidal ideation. Daniel-Calveras and colleagues [4] also found that older UYRs tend to be at a higher risk for mental disorders, which was also found in this study, with most of the participants reporting suicidal ideation being between 16 and 19 years old.

### Implications

Using ML methods, this study demonstrates their suitability for classification and out-of-sample prediction of at-risk cases and their ability to incorporate the highly individual problem constellations presented by a large sample of UYRs. Building on the findings of this study, especially regarding the link between suicidal ideation and PTSS, trauma load, and depressive symptomology, a broader implementation of mental health screenings for UYRs, using tools like *porta*, could improve the detection of at-risk cases. With ML approaches enabling individualized predictions, low threshold screening tools like *porta* could be potentially improved by implementing ML approaches, in regards to their ability to detect at risk-cases early. Targeting these at-risk cases, interventions should also focus on social factors, possibly building on family members in the country of origin.

Following up on the findings of this study, future research should further investigate the link between symptoms of mental health disorders, namely PTSS and depression and suicidal ideation and suicidal behavior, as well as sociodemographic and social factors. Next to the UYRs specific social context also focusing on biological factors could help to better understand the individual problem constellations leading to suicidal ideation. Such research could benefit from ML approaches and their ability to include a multitude of factors.

The significant difference in reported past attempts and suicidal ideation within the training and test samples also warrants further examination. Potentially relevant factors like trust and cultural background should be further investigated in regards to their influence on the willingness to disclose mental health struggles, to improve assessment modalities. In this context, investigating the relationship between postmigration stressors and mental health as well as their potential influence on trust in healthcare professionals could provide meaningful insights. Incorporating UYRs’ perspectives on screening modalities with the aim to create more culturally sensitive tools, might further improve the assessment of mental health symptoms and early detection of at-risk cases. Including ML approaches in future research could be beneficial as their potential to represent nuanced relations within the data offers a useful approach to a more concise understanding of the individual factor constellations, influencing the willingness to report as well as suicidal ideation itself. Implementing an ensemble method could further improve accuracy when trained on enough data. When using ML approaches, researchers should also focus on ethical implications such as false positives, especially when implementing ML into screening tools, as classifiers are prone to misclassification if trained on small training sets or biased data. In the domain of ML and artificial intelligence (AI), particularly in applications such as suicide prediction, it is imperative to establish robust guidelines that delineate the extent to which these technologies can influence decision-making and determine the threshold at which human validation and final decisions are mandated. The involvement of relevant professional organizations is crucial in this context; they are tasked with regulating the use of ML and AI to ensure both safe and ethically justifiable applications in the future.

### Limitations

There are some limitations that should be considered. One limitation regarding the interpretability of this study is the assessment of suicidal ideation. In the training sample, only a portion of adolescents were screened for past attempts, hence the relevance of this predictor must be interpreted with caution. However, the classifier performance shown with the test sample underlines the importance of past attempts as a predictor for suicidal ideation. A major limitation of the assessment of suicidal ideation is the usage of different assessment modalities. Although two different assessment tools were used to measure suicidal ideation and past attempts, the operationalization via binary coding of the outcome enabled its use in the ML classifiers. But the results must be interpreted with caution, due to potential bias due introduced by the assessment setting. The willingness to disclose and therefore disclosure rates might have been affected by the assessment setting, which might have influenced data quality and biased the models. In the test set, data was assessed only via self-report questionnaires and UYRs were assisted by youth welfare facility staff not necessarily trained for psychological assessment, which could have influenced the reliability of the assessment [19]. Therefore, using the same assessment modalities in training and test sets would rule out potential bias introduced by differing assessment methods and settings. Simultaneously, the utilization of different instruments and data collection methods reflects the heterogeneity of approaches across various institutions, thereby granting our findings substantial external validity and generalizability with real-life relevance. Another issue highlighted by the significant differences between training and test set is class imbalance, which occurs when one of the two classes is overrepresented in the data, something that naturally occurs when dealing with prevalence data [14, 39]. Classifiers trained on imbalanced datasets tend to recognize better the majority class, which usually is the healthy class [39]. Methods to correct for class imbalance have been criticized for potential overfitting and producing ill-calibrated models [39]. Within this study no correction for class imbalance was performed to adhere to the found prevalence and to avoid introducing bias into the analysis. The training sample used in this study is rather large compared to other studies concerning UYRs [9], and the out-of-sample validation of the classifiers yielded promising results, but larger datasets could improve the generalizability of the results.

It is also important to note that no causality can be assumed based on the results of this study due to the cross-sectional design, and further research is necessary to investigate potential causality between the found risk factors and suicidal ideation in longitudinal models. Lastly, non-suicidal self-injury (NSSI) was not assessed for the training set, so it was not represented in the analyses. NSSI often occurs alongside suicidal ideation and suicide attempts, and its role in suicidal ideation and suicide attempts amongst UYRs should be investigated in future studies [44, 45].

## Conclusion

In conclusion, this study provides the first insights regarding the prevalence and risk factors for suicidal ideation amongst UYRs, underlining the increased risk for suicidal ideation for UYRs with PTSS and depressive symptoms and provides relevant information for youth welfare facility staff and healthcare professionals. It further demonstrates utility of ML approaches for psychological research, demonstrating benefits and caveats especially regarding suicidality, and encourages their use in further research regarding risk assessment.

## Data Availability

No datasets were generated or analysed during the current study.
